# Epigenetic modifications of the glucocorticoid receptor gene are associated with the vulnerability to psychopathology in childhood maltreatment

**DOI:** 10.1038/tp.2015.63

**Published:** 2015-05-26

**Authors:** K M Radtke, M Schauer, H M Gunter, M Ruf-Leuschner, J Sill, A Meyer, T Elbert

**Affiliations:** 1 Clinical Psychology and Behavioral Neuroscience, Department of Psychology, University of Konstanz, Konstanz, Germany; 2 Evolutionary Biology and Zoology, Department of Biology, University of Konstanz, Konstanz, Germany

## Abstract

Stress, particularly when experienced early in life, can have profound implications for mental health. Previous research covering various tissues such as the brain, suggests that the detrimental impact of early-life stress (ELS) on mental health is mediated via epigenetic modifications including DNA methylation. Genes of the hypothalamic–pituitary–adrenal axis—in particular, the glucocorticoid receptor (*hGR*) gene—stand out as key targets for ELS. Even though the link between *hGR* methylation and either ELS or psychopathology is fairly well established, the mutually dependent relationships between ELS, DNA methylation and psychopathology remain to be uncovered. The specific psychopathology an individual might develop in the aftermath of stressful events can be highly variable, however, most studies investigating *hGR* methylation and psychopathology suffer from being limited to a single symptom cluster of mental disorders. Here, we screened volunteers for childhood maltreatment and analyzed whether it associates with *hGR* methylation in lymphocytes and a range of measures of psychological ill-health. *hGR* methylation in lymphocytes most likely reflects methylation patterns found in the brain and thus provides valuable insights into the etiology of psychopathology. We find the interaction between childhood maltreatment and *hGR* methylation to be strongly correlated with an increased vulnerability to psychopathology providing evidence of epigenome × environment interactions. Furthermore, our results indicate an additive effect of childhood maltreatment and *hGR* methylation in predicting borderline personality disorder (BPD)-associated symptoms, suggesting that the combination of both ELS and DNA methylation that possibly represents unfavorable events experienced even earlier in life poses the risk for BPD.

## Introduction

Childhood maltreatment affects the development of mental health in different ways. For example, the experience of childhood adversities significantly increases the risk of developing multiple psychopathologies including major depressive disorder, borderline personality disorder (BPD), anxiety disorders and substance abuse.^1, 2, 3, 4^ Compared with non-maltreated individuals, psychiatric patients with a history of childhood maltreatment are characterized by an earlier disease onset, greater symptom severity, more comorbidities and poorer responses to many first-line treatments.^5, 6, 7^ Teicher and Samson^7^ have suggested that biological determinants such as epigenetic modifications in stress-response systems, especially the hypothalamic–pituitary–adrenal (HPA) axis, may be the driving force behind the development of these childhood maltreatment-induced disorders.

**Figure 1:**
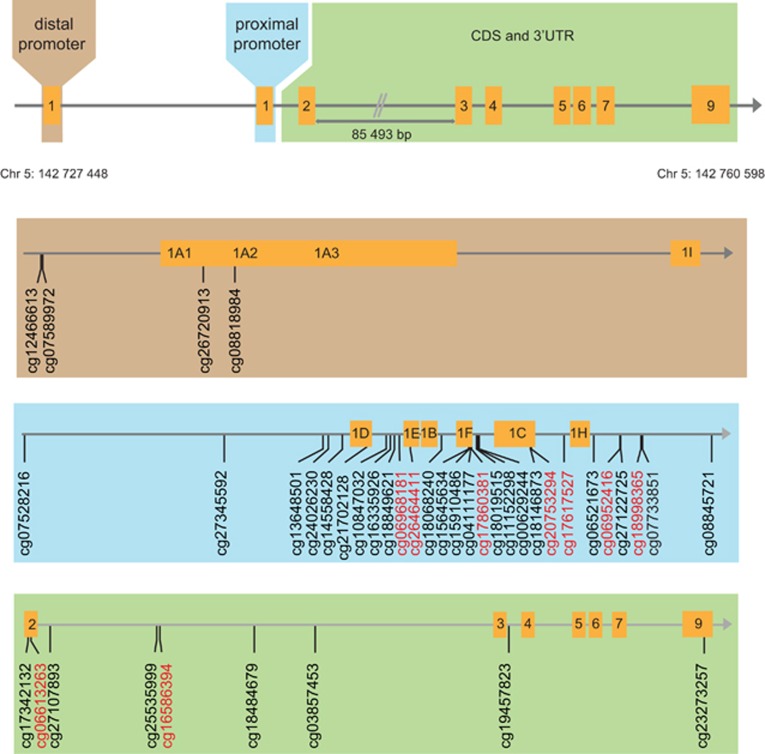
Summary of CpG sites in the glucocorticoid receptor used in our investigation. The CpG sites that were used in principal component analyses are highlighted in red font. UTR, untranslated region.

The HPA axis has a central role in translating early-life stress (ELS) into negative long-term mental health outcomes, as it is tuned by experiences occurring early in life, making it highly susceptible to ELS.^8, 9, 10^ Its dysregulation is a key feature of a range of psychopathological symptoms.^11, 12^ Both human and animal studies suggest that HPA axis function may be stably altered through aberrant epigenetic modifications resulting from ELS. In rats, offspring that have experienced ELS show an increased HPA axis response to stress^13^ as well as an increased incidence of fearful behaviors.^14^ The glucocorticoid receptor (GR) gene and its methylation status are of central relevance to this phenotype.^15^ Genes that display high levels of DNA methylation in their promoter regions tend to be less transcriptionally active.^16^ The GR initiates the feedback inhibition of the HPA axis—after binding its ligand, cortisol, it dampens HPA axis activity. The human GR (*hGR*) consists of eight coding (2–9) and one non-coding (1) exons^17^ (Figure 1). The promoter region of the gene, non-coding exon 1, consists of several alternate exons (1 A, 1I, 1D, 1E, 1B, 1F, 1C and 1H) that give rise to multiple transcripts that encode the same protein.^17^ Previous research has demonstrated that the methylation of alternate exon 1F seems to be strongly influenced by ELS. Consistent with increased HPA axis responses, alternate exon 1_7_—the murine homolog to alternate exon 1F—is hypermethylated in the brains of stressed juvenile rats, concomitant with a decrease in GR expression.^13, 15^

Correspondingly, in humans, methylation of the *hGR* promoter is associated with both ELS^18, 19, 20, 21, 22, 23^ and psychopathology. A history of childhood abuse in suicide victims—a group generally displaying high rates of psychopathology^24^—is associated with increased *hGR* methylation in brain tissue.^25, 26^ Correspondingly, in the blood of patients suffering from BPD, that is, in individuals that usually have been exposed to severe forms of abuse and neglect during development, *hGR* methylation was observed to be increased^27^ and to be positively correlated with BPD-symptom severity.^28^ On the other hand, decreased hGR-promoter methylation in both saliva and blood seems to be related to more intrusive memories and an increased risk of developing posttraumatic stress syndrome when stressors are encountered in adulthood.^25, 29^ In spite of its importance to the field, none of this previous research has resolved whether epigenetic processes that alter HPA axis function expose the individual to a higher risk of developing a psychopathology, or alternatively, whether these changes are neutral or even serve to adapt the behavior towards survival in a hostile, abusive environment. All of the aforementioned studies that investigated the contribution of *hGR* methylation to the development of psychopathology were limited to a single measurement of psychological health. Therefore, it is yet unknown how *hGR* methylation contributes to psychopathology more generally.

Here, we investigate the mutually dependent relationships between ELS, epigenetic modifications and mental health. Utilizing a multivariate approach, incorporating several psychological dimensions, we aimed to investigate whether the simultaneous occurrence of ELS and increased *hGR* methylation is accompanied by an increased vulnerability to the development of psychopathology. We predicted that childhood maltreatment would be associated with a greater range and intensity of psychopathological symptoms and that this association is modulated via DNA methylation of the *hGR* gene.

## Materials and methods

### Participants

Forty-six participants (*N*_female_=28, *N*_male_=18), aged between 11 and 21 years (median=15) were recruited, with an emphasis on including individuals that varied in the degree of childhood adversity experienced. The study cohort represents a convenience sample from the local community with an announcement that the investigation would include investigation of potential biomarkers of ELS. A subset (*N*=23) had already participated in an earlier study.^20^ Participants received a total of 30 Euros and all participated on a voluntarily basis, that is, free to quit the interview at any time without giving any reasons and without losing the reimbursement. The study was approved by the institutional review board (Ethics Committee) of the University of Konstanz.

Childhood maltreatment and psychological ill-health were assessed using structured interviews by experienced clinical psychologists. Childhood maltreatment was assessed in detail using a draft version of the KERF-I,^30^ a German version of the pediatric MACE^31^ interview with good psychometric properties, which is designed to match the needs of children.^30^ It captures the lifetime occurrence of childhood maltreatment up to age 18 in eight dimensions: parental physical abuse, parental emotional abuse, sexual abuse, witnessed physical violence toward parents, witnessed violence toward siblings, peer physical violence, physical neglect and emotional neglect.

Symptoms associated with BPD were assessed using the Borderline Symptoms Checklist-23.^32^ The Borderline Symptoms Checklist-23 was constructed according to the Diagnostic and Statistical Manual of Mental Disorders (fourth edition) criteria for BPD. It consists of 23 items that are scored from 0 (not at all) to 4 (very strong). A resulting sum-score of 64 or higher is indicative of a clinically relevant BPD-diagnosis. Symptoms of depression and anxiety dimension have been evaluated using the Hopkins Symptoms Checklist-25.^33^ Symptoms associated with oppositional defiant disorder, conduct disorder or attention deficit hyperactivity disorders were evaluated using the respective parts of the Mini-International Neuropsychiatric Interview.^34^ The perceived health-related life quality has been assessed using the KIDSCREEN-53.^35^

The Strength and Difficulties Questionnaire^36^ was administered to evaluate strength and difficulties in four dimensions. Resulting scores of ⩽15, ⩾16 or ⩾20, are classified as being ‘Normal', ‘Intermediate' or ‘Abnormal', respectively.

### DNA Methylation

Blood samples were collected immediately after completion of the interviews. Lymphocytes were isolated via a Ficoll (Sigma-Aldrich, Saint Louis, MO, USA) gradient and subjected to DNA extraction (DNeasy Blood and Tissue Kit, Qiagen, Hilden, Germany). Genome-wide analysis of DNA methylation was then conducted at the Barts and the London Genome Centre (Queen Mary University of London, London, UK). Genomic DNA (1 μg) was bisulfite converted (EZ DNA Methylation Kit, Zymo, Irvine, CA, USA) and applied to the Human Methylation 450 K array (Illumina, San Diego, CA, USA).

Samples that exhibited either abnormal methylation profiles across all CpG sites or signs of unconverted DNA based on the conversion control probes present on the array were excluded before further processing and analyses (*N*=0). The Human Methylation 450 K array includes two different bead types associated with two different chemical assays, the Infinium I and the Infinium II assay. To compensate for this, the raw data were normalized using both the R package lumi and Beta Mixture Quantile dilation.^37^ To eliminate any potential biases that may have arisen due to differences in the labeling and scanning properties of these two bead systems, color adjustment was performed through lumi. To reduce any further systematic biases, quantile normalization was used through lumi.^38^ To adjust for the probe-type bias, Beta Mixture Quantile dilation was performed on the quantile normalized data. After preprocessing, DNA methylation was assessed for all probes spanning the *hGR* gene, which are included on the Human Methylation 450 K array, identified according to their genomic positions. This set included a total of 41 probes (Figure 1).

### Statistical analyses

All statistical analyses were conducted in RStudio.^39^ Depending on the distribution, either parametric Pearson correlations or nonparametric Spearman correlations were performed. Normality was assumed if skewness was less than an absolute value of 2 and kurtosis was less than an absolute value of 7. Our analysis used a false discovery rate of 0.05 to account for multiple testing across the 41 CpG sites in the *hGR* gene.^40^ Adjusted *P*-values were computed using the Benjamini–Hochberg procedure. Our metric for a small effect size was *r*⩾0.10, for a medium effect *r*⩾0.30 and for a large effect *r*⩾0.50.^41^ Principal component analyses were performed using the psych-package.^42^ To avoid multicollinearity, the predictors were mean-scaled before computing the interaction term in multiple-regression analyses. Furthermore, the dependent variable was log-transformed, if necessary, to fulfill the assumptions about the residuals in multiple-regression analyses.

### Transcription factor binding sites

To reveal potential functional properties associated with the CpG sites included in our study, the sequence 50 bp up- and downstream of the respective CpG sites have been submitted to the Jaspar database^43^ to predict known transcription factor binding sites (TFBSs). In addition the University of California Santa Cruz genome browser^44^ was used to screen the respective genomic regions for conserved TFBSs or marks usually associated with transcriptional activity, such as H3K27 acetylation.

## Results

Subjects reported that they had experienced between one and 20 different events of childhood adversities (Table 1). The majority of the subjects displayed subclinical values with respect to psychopathological symptoms with some presenting clinically relevant symptoms of anxiety and/or depression. The psychometric measurements obtained appeared to be randomly distributed among the participants, with no specific symptoms clustering within certain individuals (Supplementary Figure S1).

### Relationship between *hGR* methylation, childhood abuse and psychological ill-health

We investigated DNA methylation in 41 CpG sites associated with the *hGR* gene in lymphocytes (Supplementary Table S1). Neither of the investigated psychometric measurements nor childhood maltreatment was associated with average methylation of all investigated CpGs that were distributed across the whole *hGR* (data not shown). However, we observed statistically significant correlations between the methylation of two specific CpG sites located in the promoter of the *hGR* gene, and stress- or psychological ill-health-related measurements.

**Figure 2:**
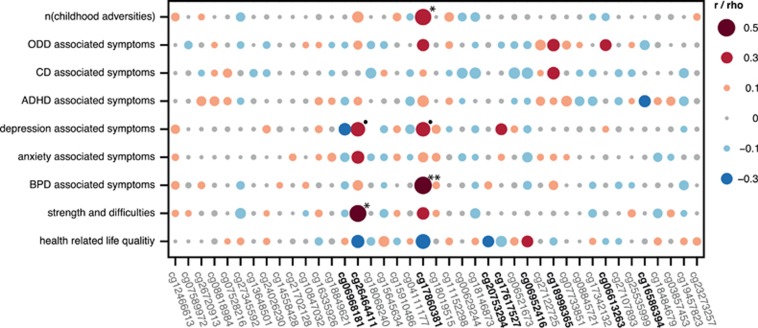
Correlations between individual CpG sites and stress exposure or psychological health. The strength of the correlations are depicted by a color gradient, ranging from far blue (negatively correlated) to far red (positively regulated). The size of the dots represents the effect size, that is, the larger the dot, the greater the effect size. The CpG sites that were used in principal component analyses are highlighted in black font. ADHD, attention deficit hyperactivity disorder; BPD, borderline personality disorder; CD, conduct disorder; ODD, oppositional defiant disorder; adj ^·^*P*⩽0.1; adj *P⩽0.05; adj **P⩽0.01; adj *P*, adjusted *P-*value.

We identified a positive correlation between methylation of cg17860381 (located in the alternate exon 1F) and childhood maltreatment, as indicated by the statistically significant correlations with the number of experienced childhood adversities (Figure 2, Supplementary Table S2). Interestingly, methylation of the same CpG site also displayed a highly significant positive correlation to BPD symptoms (Figure 2, Supplementary Table S2). In addition, methylation of cg17860381 was positively correlated to depression symptoms to a degree that approached statistical significance (Figure 2, Supplementary Table S2).

Methylation of a second CpG site, cg26464411, was also positively correlated with two measurements associated with psychological health. The correlation with depression symptoms was statistically significant, while the correlation with behavioral strength and difficulties approached statistical significance (Figure 2, Supplementary Table S2).

Apart from the large effect sizes displayed in the correlations between the methylation of cg17860381 with BPD-associated symptoms and between the methylation of cg26464411 and behavioral strengths and difficulties, all of the remaining aforementioned correlations displayed medium effect sizes. Especially for small samples, effect size may be the reference of choice, as the *P*-value heavily depends on sample size and thus true effects are prone to be missed.^45^ Although not being statistically significant by means of *P*-values, both methylation of cg17860381 and of cg26464411 negatively correlated with health-related life quality at medium effect sizes. Furthermore, oppositional defiant disorder-associated symptoms and behavioral strength and difficulties were positively associated with cg17860381 methylation, while anxiety-associated symptoms correlated with cg26464411 methylation, each with medium effect sizes. Along the same lines, considering effect size alone, further associations with medium effect sizes were observed. The methylation at seven additional CpG sites correlated with psychological health-related measurements including health-related life quality and symptoms associated with oppositional defiant disorder, conduct disorder, attention deficit hyperactivity disorder, depression, anxiety and BPD (Figure 2).

To further investigate the relationship between childhood abuse, methylation of the *hGR* gene and psychological ill-health, we performed two principal component analyses (PCA). These comprised either variables relating to DNA methylation or psychological health. In PCA, the first principal component (PC) accounts for the largest proportion of the variance created by the included variables. Therefore, each of the obtained first PCs was used in a subsequent multiple-regression analysis. All psychological health-related variables correlating with the methylation of at least one CpG site with at least medium effect size and the corresponding CpG sites were considered for PCAs. Using this threshold, all of the elevated psychometric variables (*n*=8) and nine CpG sites were included in the PCAs. The first PCs relating to either psychological health or DNA methylation explained 61.7% or 20.5% of the variance, respectively. For the PC comprising psychological ill-health, an exploratory factor analysis identified absolute factor loadings ranging between 0.68 and 0.88 with maximum loadings for depression- and anxiety-associated symptoms (Supplementary Figure S1a). For *hGR* methylation, absolute factor loadings ranged between 0.11 and 0.59 with the highest loading for cg17860381 (Supplementary Figure S1c).

**Figure 3:**
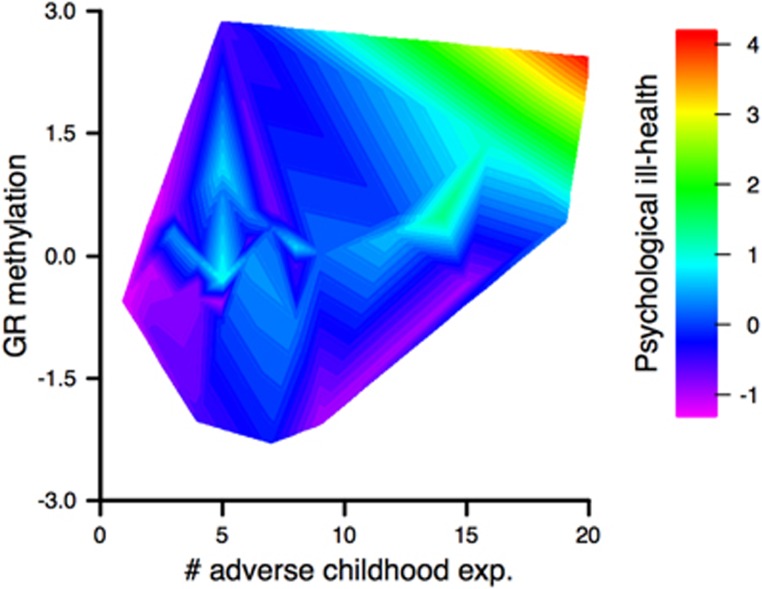
Relationship between psychological ill-health, childhood abuse and *hGR* methylation. The *hGR* methylation and psychological ill-health are represented by the first principal components of a principal component analyses summarizing nine CpG sites in the glucocorticoid receptor gene and all evaluated psychometric measurements, respectively. The color gradient ranging from purple to red represents the level of psychological ill-health, that is, purple for healthy and red for unhealthy. exp., experiences; *hGR*, human glucocorticoid receptor.

Next, we performed a linear model using the first PC comprising psychological ill-health as outcome and the number of experienced childhood adversities, the first PC comprising *hGR* methylation as well as their interaction as predictors. The model explained 63.2% (F(5,36)=12.4, *P*⩽0.001) of the variance. Both of the predictors as well their interaction impacted psychological health significantly (*β*_*hGR* methylation_=0.3, *P*_*hGR* methylation_⩽0.05, *β*_*n* (childhood adversities)_=0.3, *P*_*n* (childhood adversities)_⩽0.05, *β*_interaction_=0.5, *P*_interaction_⩽0.001, Figure 3). To correct for effects potentially arising from sex or age,^46^ these variables were also included in the model. Neither sex nor age seemed to affect psychological health (*β*_sex_=0.1, *P*_sex_>0.1, *β*_age_=−0.1, *P*_age_>0.1).

### Interaction of childhood adversities and methylation of cg17860381 in the development of BPD

**Figure 4:**
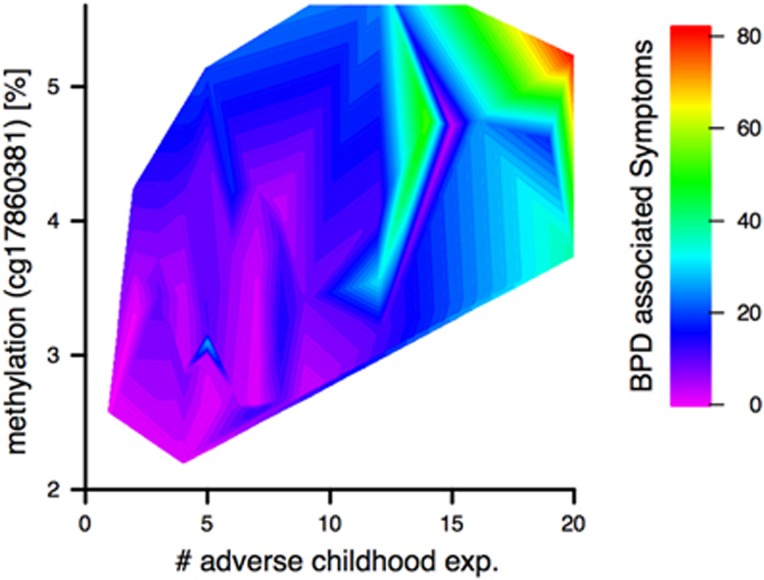
Relationship between methylation of cg17860381, adverse childhood experiences (exp.) and BPD-associated symptoms. The color gradient ranging from purple to red represents the level of BPD-associated symptoms, that is, purple for low levels and red for high levels. BPD, borderline personality disorder.

As both BPD-associated symptoms and childhood adversities correlated significantly with methylation at cg17860381, we hypothesized that the simultaneous occurrence of both facilitates the development of BPD symptoms. To further investigate a potential interaction between methylation at cg17860381 and childhood adversities, influencing the subsequent development of BPD symptoms, we performed a linear model using these two variables and their interaction as predictors for BPD symptoms. To guarantee heteroscedasticity of the model residuals, BPD-associated symptoms were log-transformed before the analysis. The model accounted for 52.6% (F(5,40)=8.87, *P*⩽0.001) of the variance in BPD symptoms. Under this model, both of the predictors impacted BPD symptoms significantly (*β*_cg17860381_=0.5, *P*_cg17860381_⩽0.01, *β*_*n* (childhood adversities)_=0.5, *P*_*n* (childhood adversities)_⩽0.01, Figure 4), whereas their interaction did not exert contribution to BPD symptoms that reached statistical significance (*β*_interaction_=−0.2, *P*_interaction_⩽0.1). To correct for effects potentially arising from sex or age, we also included those as variables in the model. While the former did not influence BPD symptoms, there was a statistical trend towards an influence of age on BPD symptoms (*β*_sex_=−0.2, *P*_sex_>0.1, *β*_age_=0.2, *P*_age_⩽0.1). An investigation of the genomic location did not reveal the presence of a TFBS.

## Discussion

ELS is known to exert a lifelong detrimental influence on mental health, and previous studies suggest that this is associated with methylation of the glucocorticoid receptor (*hGR*) gene in various tissues.^15, 19, 26^ However, to date, no study has explicitly tested for a relationship between ELS and both *hGR* methylation and psychopathology. Moreover, very few studies include analyses of psychopathology in multiple dimensions. Rather than discovering the etiology of particular psychopathological states, we aimed to unravel the threats posed by ELS to psychological well-being as a general concept. Therefore, in this study, we analyzed psychological ill-health from several perspectives, including testing for symptoms associated with oppositional defiant disorder, conduct disorder, attention deficit hyperactivity disorder, depression, anxiety or BPD. In addition, we focused on the perceived life quality, functioning and strength and difficulties, as they are associated with various categories of disorders, such as the ones mentioned above. By using a combined approach that incorporated PCA and multiple-regression analysis, we identified a strong relationship between childhood maltreatment, methylation of the *hGR* gene in lymphocytes and psychological ill-health. Moreover, we identified an additive effect of childhood maltreatment and cg17860381 methylation in the hGR promoter on the development of BPD-associated symptoms.

In addition to an additive effect of both *hGR* methylation and childhood maltreatment, the interaction of these two variables predicted the intensity of psychopathological symptoms. Thus, the combination of childhood maltreatment with h*GR* methylation by means of the composite methylation of nine CpGs spanning the entire *hGR* is associated with impaired mental health, that is, we observed an epigenome × environment effect. Given the substantial evidence for gene × environment effects on psychological well-being, we anticipated a similar epigenome × environment effect, even though—to the best of our knowledge—it had not been demonstrated yet. Although the impact of methylation at single or few CpG sites on gene transcription is debated, in the case of the glucocorticoid receptor, site-specific correlations between DNA methylation and gene expression and/or ELS have been repeatedly reported.^19, 25, 26, 47^ Moreover, different directions of correlations between childhood abuse and DNA methylation have been identified for different regions of the hGR promoter.^25^ To avoid canceling out of opposing effects, we chose a PCA-based approach rather than averaging several CpG's methylation to gauge *hGR* methylation. Indeed, we did not find an effect for average methylation. For BPD-associated symptoms, we focused on its relationship between DNA methylation at a single CpG site—cg176860381—and childhood maltreatment. This relationship seems to be simpler, as it shows a strong additive effect of childhood maltreatment and cg176860381 methylation, whereas the interaction of those two predictors did not impact the intensity of BPD-associated symptoms. Interestingly, cg17860381 also seems to have an important role in the above-described relationship between childhood maltreatment, *hGR* methylation and psychopathological symptoms, as it exerted the highest influence on the joint principal component summarizing *hGR* methylation. This CpG site is part of the alternate exon 1F, which has been previously found to be associated with ELS or psychopathology.^15, 18, 19, 20, 21, 22, 23, 27^ Due to the location of cg17860381 in the proximal promoter of *hGR*, it is plausible that methylation at this site may influence the dynamics of *hGR* expression, although it is not located at the TFBSs recently implicated in the epigenetic regulation of *hGR*-expression^15^ nor could we detect another TFBS nearby. Thus the functional implications of cg17860381 methylation need to be further investigated. In addition, future studies should also evaluate other epigenetic mechanisms such as histone modifications or gene expression to gain a more cohesive picture. Our results suggest that *hGR* methylation in lymphocytes—either evaluated at a single CpG site or as the joint profile of several CpGs—represents certain environmental conditions setting these individuals at a higher risk of developing psychopathology. Indeed, *hGR* methylation in blood or brain tissue was shown to associate with exposure to prenatal stress,^20, 21^ a familial history of posttraumatic stress disorder^48^ or childhood stress.^18, 22, 26^ However, the exact causes of *hGR* methylation need to be further investigated. Meaney and Szyf^49^ suggested that hippocampal GR methylation results from a lack of tactile stimulation. Already Harlow proved that somatosensory deprivation can cause social–emotional disorders^50^ that were later suggested to be mediated by impaired cerebellar development.^51^ BPD patients have frequently been exposed to childhood neglect,^3^ which usually associates with somatosensory deprivation. Accordingly, BPD was found to be associated with decreased cerebellar vermis size^52^ as well as with reduced balance skills,^53^ which both suggest functional cerebellar impairment. Thus, the differential cg17860381 methylation in our sample might reflect childhood somatosensory deprivation. Accordingly, we suggest the course of early neglect with somatosensory deprivation and later childhood abuse as the toxic combination that promotes BPD symptoms.

Several other studies analyzed childhood maltreatment and *hGR* methylation. Perroud *et al.*^18^ showed that childhood sexual abuse correlates with *hGR* methylation in blood drawn from patients suffering from either BPD or major depressive disorder. However, this study lacked healthy controls, thus attributing the observed differences in DNA methylation only to the advent of childhood abuse. To the best of our knowledge, ours is the first study to provide a combined contribution of both ELS and methylation in a regulator gene of the HPA axis to psychological health. Three other studies have combined the analysis of ELS, *hGR* methylation in blood with phenotypic characterizations, showing that *hGR* methylation is associated with both ELS and either lower birth weight^21^ or altered cortisol levels,^19, 22^ two features that are predictive for psychopathology.

The methodologies used by our study present some limitations. Our data are correlational in nature and thus cannot prove a causal relationship between child maltreatment and methylation patterns or decreased psychological well-being. In addition, due the limited sample size, generalizations to the general population must be regarded with caution. The brain constitutes the most prominent organ into which differential DNA methylation, and hence differential gene expression, affects behavior. As epigenetic modifications occur in a tissue-specific manner, it remains unclear at this point whether DNA methylation measured in blood reflects DNA methylation patterns in the brain. However, research conducted on the effects of ELS on the methylation of the alternate promoter 1F in both blood^20^ and brain tissue^26^ points in the same direction, that is, ELS being associated with increased DNA methylation. In addition, a recent meta-analysis reported methylation patterns in blood and brain tissue to be highly correlated.^54^ Accordingly, individual differences in *hGR* methylation in peripheral tissue, that is, saliva, were found to associate with differential brain activity.^29^ Together, these findings suggest that similar patterns of *hGR* methylation may be present across tissues and that our results are likely to reflect DNA methylation patterns present in the brain.

## Conclusions

Our results indicate an increased vulnerability to develop a psychopathology in general and BPD in particular, if childhood maltreatment is combined with increased methylation of the *hGR* gene, as exemplified here in lymphocytes. Remarkably, rather than being exposed to extreme forms of stress, our participants were exposed to only moderate levels of childhood maltreatment. Strengthened by the inclusion of epigenetic markers, we emphasize the threats of moderate stress to psychological well-being. In conclusion, we highlight a plausible molecular mechanism by which ELS might translate into undesirable consequences on mental health later in life, and in doing so, greatly strengthen the utility of *hGR* methylation in peripheral tissues such as lymphocytes as a potential diagnostic marker to identify whether victims of childhood maltreatment are at risk of developing severe psychopathological symptoms later in life.

## Figures and Tables

**Table 1:** Psychometric measurements

	n	*Mean (±s.d.)*	*Range*	*Max score possible*	*Clinical cut-off*	*Clinically diagnosed/classification*
*n* (childhood adversities)	46	7.4 (4.9)	1–20	35	NA	NA
Perceived health-related life quality	43	201.4 (28.8)	129–239	260	NA	NA
BPD-associated symptoms	46	12.1 (15.0)	0–81.6	89	64	1 (2.1%)
Strength and difficulties	45	11.9 (5.6)	1–26	40	Intermediate=16, Abnormal=20	Normal=32 (71.1%), Intermediate=8 (17.8%), Abnormal=5 (11.1%)
Anxiety-associated symptoms	46	6.0 (5.7)	1–27	30	8	15 (32.6%)
Depression-associated symptoms	46	9.4 (8.7)	0–37	45	12	14 (30.4%)
ADHD-associated symptoms	45	4.6 (3.8)	0–18	18	12	1 (2.1%)
CD-associated symptoms	46	1.0 (1.7)	0–8	15	3	4 (8.7%)
ODD-associated symptoms	46	1.8 (2.0)	0–8	8	4	5 (11.0%)

Abbreviations: ADHD, attention deficit hyperacitvity disorder; BPD, borderline personality disorder; CD, conduct disorder; NA, not applicable; ODD, oppositional defiant disorder.
