# Supplementary material for: Epigenetic modifications of the glucocorticoid receptor gene are associated with the vulnerability to psychopathology in childhood maltreatment
Source: Transl Psychiatry. 2015 May 26;5(5):e571–. doi: 10.1038/tp.2015.63 (PMC4471294; doi:10.1038/tp.2015.63)
Supplement: Supplementary Figure S1 [file tp201563x1.pdf]

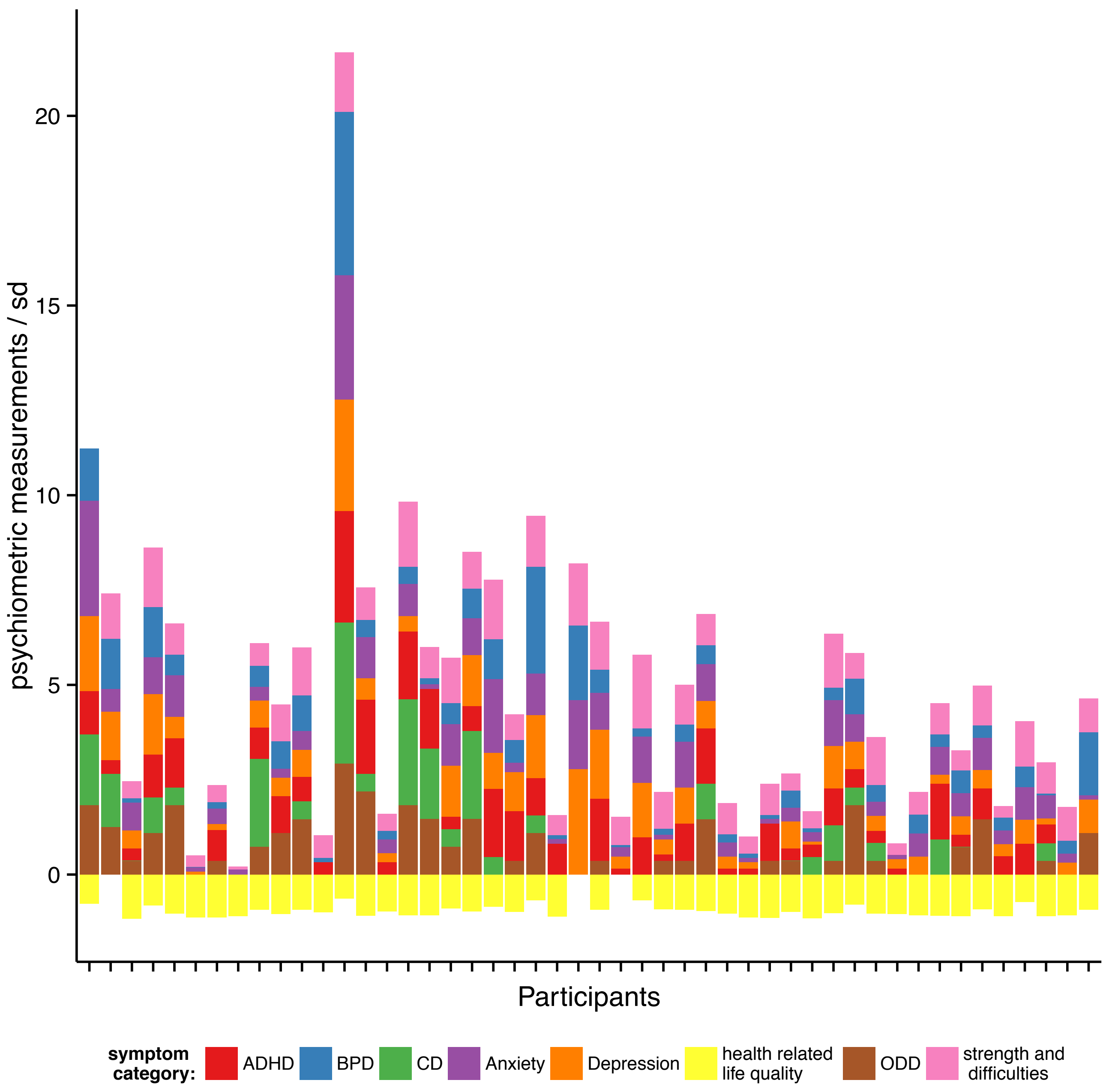
psychiometric measurements / sd
0
5
10
15
20
Participants
symptom category:
ADHD
BPD
CD
Anxiety
Depression
health related life quality
ODD
strength and difficulties
